# Prevalence and Correlates of Depressive Symptoms Among High School Students in Hanover, Jamaica

**DOI:** 10.1100/tsw.2007.104

**Published:** 2007-05-01

**Authors:** Olaniyi J. Ekundayo, Joana Dodson-Stallworth, Michelle Roofe, Inmaculada B. Aban, Mirjam C. Kempf, John E. Ehiri, Pauline E. Jolly

**Affiliations:** ^1^ Department of Epidemiology, School of Public Health, University of Alabama at Birmingham, USA; ^2^ Division of HIV/AIDS Prevention, National Center for HIV, STD, TB Prevention Centers for Disease Control and Prevention, Atlanta, GA, USA; ^3^ North East Regional Health Authority Ocho Rios, St Ann, Jamaica; ^4^ Department of Biostatistics, School of Public Health, University of Alabama at Birmingham, USA; ^5^ Department of Maternal and Child Health, School of Public Health, University of Alabama at Birmingham, USA

**Keywords:** depressive symptoms, sexual experience, maternal affection and support

## Abstract

The objective of this study was to determine the prevalence of depressive symptoms in Jamaican adolescents and examine its association with individual and family factors. We used an abbreviated form of the Beck's Depression Inventory II (BDI-II) to assess depressive symptoms among 748 students, attending public high schools in the parish of Hanover Jamaica. In the analysis, we classified adolescents with scores in the upper quartile of the depressive symptom score as having depressive symptoms. Multivariate logistic regression was used to determine the predictors of depressive symptoms. 14.2% of participants reported depressive symptoms. There was association between engagement in sexual activity [Odds Ratio (OR) = 1.61, 95% Confidence Interval (CI) = 1.02-2.51], parental monitoring of adolescent activity (OR=2.04, 95%CI=1.33 -3.12), maternal affection and support (OR= 4.07, 95%CI= 2.62-6.33), and paternal affection and support (OR= 1.58, 95%CI= 1.05-2.39) with self reported depressive symptoms at the bivariate level. In the final model, depressive symptoms was associated with perceived lack of maternal affection and support (OR= 4.06, 95%CI= 2.61-6.32) and showed marginal association with being sexually experienced (OR= 1.59, 95%CI= 1.00-2.52). As most homes are female-headed, establishing support systems for the mother to take care of their adolescent children may decrease the odds of depressive symptoms. Sexually experienced adolescents may require screening for depression. Further research is required to fully explore all factors that could predispose Jamaican adolescents to depression.

### INTRODUCTION

Adolescent depression is a highly prevalent condition that is often under-recognized by families and physicians alike. Depression may be viewed as normal mood swings typical of the adolescent stage[1]. Research findings indicate that depression may in fact occur more at this age than at any other life stage[1,2,3]. As a result, adolescent depression has become a major public health issue in recent times, with researchers calling for interventions[2,3]. Of concern is the fact that a good proportion of adolescents exhibit depressive symptoms that are sub-threshold for the diagnosis of major depressive disorder[3].

Current researches from western countries have reported on factors associated with depressive symptoms among adolescents. Risk factors reported include individual variables such as initiation of sexual intercourse[4,5,6], increasing age[3,7], and gender[3,4], family factors such as parental low socioeconomic status[8,9], family structure[9,10], and lack of parental love and support[2,8]. Other factors include pubertal timing[5,11], poor grades and academic progress in school[10], trouble with peers[12], substance use[1,7], and other psychosocial measures such as poor self esteem[13].

Studies have shown that engaging in sex places adolescents, especially girls at risk for depression[4,5,6,14]. Adolescent girls engaging in sexual activity have been reported to experience depressive symptoms more than boys with similar behavior[14,15]. Tubman et al reported that sexually active adolescents displayed more depressive symptoms than their sexually abstinent peers[14]. In their analysis of the National Longitudinal Survey of Adolescent Health, Rector et al[4] reported that sexually active girls were more than three times more likely to be depressed than girls who were not sexually active. In the same study, boys who were sexually active were twice as likely to be depressed compared to those who were not sexually active[4]. Furthermore depressed adolescents are more likely than any other youth to engage in unsafe sexual practices and other risky behaviors[6,7].

There are no studies designed to identify correlates of depressive symptoms among rural adolescents in Jamaica. Less is also known about the influence of factors such as engagement in sexual activity and parental support on adolescent depression. In this study, we examined the effect of individual and family context variables on depressive symptoms among rural adolescents in Jamaica. We hypothesized, based on research findings in the literature that depressive symptoms will be positively associated with adolescent sexual experience and lack of parental support.

### METHODS

#### Participants

The participants in this survey were part of "The Hanover Teen Study" conducted in the summer of 1998 to identify factors associated with adolescent sexual activity in Jamaica[15]. Briefly the study was a cross-sectional survey of high school students attending different types of public high schools in the parish of Hanover, Jamaica, and was designed to identify factors associated with adolescent sexual activity in this parish. Schools in the parish were grouped based on the type of admission into the schools. The schools selected for participation in this study were comprehensive high schools (formerly new secondary schools (group 1), and technical and agricultural high school (group 2). Students who perform well in the National Assessment Examination taken at the end of elementary school attend academically rigorous secondary high school and technical high schools, which prepare them for college. On the other hand, admission to new secondary schools (now comprehensive high schools) providing a mixture of academic and vocational training is gained from feeder schools and those who did not perform well on the National Assessment Examination with little chance of continuing their education beyond the secondary level[16]. Seven hundred and eighty eight students attending the selected schools participated in the survey.

### Procedure

The protocol for this study was reviewed and approved by the institutional review board (IRB) of the University of Alabama at Birmingham and the Ethics Committee, Ministry of Health, Jamaica prior to implementation. Written informed consent from parents and the students were also obtained.

### Instruments and Measures

#### *Socio-demographics*

Socio-demographic data were collected on variables such as current age at time of survey and gender. We obtained information on adolescents' sexual experience based on their response to the question "Have you ever had sex: (Yes/No)?" In this study, sex was defined as a penis entering the vagina. Given the sensitive nature of this question and other questions on depression, they were asked towards the end of the survey, after questions about the adolescent's personal and family characteristics had been answered.

#### *Family Context Variables*

Family structure was measured according to whether the adolescent live in intact two-parent family or not. We defined intact family as married biological parents living in the same household with the adolescent.

Parental monitoring and supervision of adolescent activities was measured utilizing three items to determine the extent to which parents know their child's whereabouts, how much interest they show in who they spend time with, and what the adolescent does during their free time. An example of this item is "my parents know where I go after school and on weekends". Response options range from 1 (all the time) to 5 (none of the time). A composite variable based on the sum of these three items was constructed to determine the level of monitoring of adolescents' activities by the parents. A high score on these scales is considered as lack of parental monitoring/supervision (Cronbach's alpha = 0.54)

Perception of parental affection and support was measured using a four-item measure (each for the mother and the father). A sample item included the following phrase: "I feel loved by my mother/father." Response options to these questions range from 1 (all the time) to 4 (none of the time). Adolescents who were not living with their biological parents were asked to substitute "primary female or male guardian" for "mother/father". Scores were combined separately for each parent to evaluate the level of affection between the adolescent and each of their biological or surrogate parents. High scores indicate lack of maternal affection and support (Cronbach's alpha = 0.74) and/or lack of paternal affection and support (Cronbach's alpha = 0.83).

#### *Outcome Variable*

The depressive symptom measure consisted of three somatic-affective and psychological symptoms questions (sadness, tiredness, and suicidal thoughts) modified from Beck's Depression inventory II (BDI II) (18) BDI II measures the severity of self-reported depression in adolescents and adults. Beck et al factor analyzed the BDI-II responses of 120 undergraduates and identified two dimensions for self-reported depression symptoms[17]. The first factor was a somatic-affective dimension represented by salient (≥ 35) loadings for somatic symptoms such as tiredness and loss of energy, and affective symptoms such as sadness and crying. The second factor was composed of psychological symptoms, such as suicidal thoughts and worthlessness. Several affective symptoms such as sadness and crying switched between factors[17]. Items were selected to represent each of the dimensions based on their high loadings on factor analysis and ease of response by adolescents. Respondents were asked to describe themselves in relation to these symptoms in the past two weeks, including the day of the interview. Each symptom is rated on a 4-point scale ranging from 0-3. The maximum score was 9. The internal consistency of the scale for this sample of Jamaican adolescents was 0.79, as measured by Cronbach's alpha statistics. We defined those who scored in the upper quartile of the distribution of scores as having depressive symptoms[18]. This was done to distinguish depressive symptoms from normal mood swings during adolescence. It is possible that adolescents who reported depressive symptoms may have met clinical diagnostic criteria for clinical depression; however, this study did not attempt to link self-reported depressive symptoms with the diagnosis of clinical depression[2].

### *Sample*

The data generated from this study were analyzed for missing responses using the Statistical Package for the Social Sciences (SPSS) frequency procedure. Out of the original 788 participants, we excluded those with incomplete data (n = 40) on key predictor variables such as gender and engagement in sexual activity from the analysis. For the 748 individuals remaining in the analysis, missing data appeared to be randomly distributed across gender groups. We replaced missing values for continuous variables or for individual items summed to produce composite scores by substituting the mean response given by respondents of the same gender. The range of missingness for continuous explanatory variables for this specific aim was between 0.7% and 3.3%. The largest (3.3%) is attributed to one of the measures of paternal affection and support "I feel loved by my father".

### Statistical Analysis

Frequency distributions were computed for selected socio-demographic characteristics. Mean values and standard errors of the mean (SEM) were calculated for continuous variables. We used Pearson's chi-square and t-tests to test the associations of these characteristics and depressive symptoms. We then proceed to perform logistic regressions between the outcome variable and each of the explanatory variables (bivariate) and between the outcome variable with all explanatory variables in the model (multivariate) to estimate crude and adjusted odds ratio (OR) with 95% confidence intervals (CI). We presented the results as unadjusted and adjusted ORs with 95% CI, for the assessment of the stability of the association when all covariates were adjusted for in the model. In the final model, variables were eliminated using stepwise and backward strategy with a 10% cut-off margin.

All tests were two-tailed, and a p-value of 0.05 or less was considered as statistically significant. The Nagelkerke's $R^2$ statistic which measures how much variation in the dependent variable the model accounts for was used in this study to assess the strength of the association predicted by the final model[19]. Statistical analysis was conducted using SPSS version 14 (SPSS Inc., Chicago, Illinois).

## RESULTS

Table 1 represents the demographic and family characteristics of the sample. Using a cut-off score in the upper quartile of the abbreviated BDI-II, about 1 in 7 participants reported depressive symptoms.

**TABLE 1**
**Individual and family characteristics of participants by depressive symptoms**

| Characteristic, N (%) or mean ± SEM[1] | All N= 748 | Depressive symptoms Yes N=106 (14.2) | Depressive symptoms No N=642 (85.8) | p-value |
|---|---|---|---|---|
| Gender | | | | 0.10 |
| Boys | 264 (35.3) | 30 (28.3) | 234 (36.4) | |
| Girls | 484 (64.7) | 76 (71.7) | 408 (63.6) | |
| Age in years at time of survey | | | | 0.33 |
| 14 | 10 (1.4) | 3 (2.8) | 7 (1.1) | |
| 15 | 189 (25.3) | 23 (21.7) | 166 (25.9) | |
| 16 | 302 (40.4) | 47 (44.4) | 255 (39.7) | |
| 17 | 128 (17.1) | 16 (15.1) | 112 (17.4) | |
| 18 | 68 (9.1) | 7 (6.6) | 61 (9.5) | |
| 19 | 51 (6.8) | 10 (9.4) | 41 (6.4) | |
| Sexually experience | | | | 0.04 |
| Yes | 469 (62.7) | 76 (71.7) | 393 (61.2) | |
| No | 279 (37.3) | 30 (28.3) | 249 (38.8) | |
| Family structure | | | | 0.08 |
| Living with both biological parents | 312 (41.7) | 36 (34.0) | 276 (43.0) | |
| Not living with both biological parents | 436 (58.3) | 70 (66.0) | 366 (57.0) | |
| Parental monitoring[2] | 5.7 ± 0.1 | 6.3 ± 0.2 | 5.6 ± 0.1 | 0.001 |
| Maternal affection and support[3] | 7.4 ± 0.1 | 9.4 ± 0.3 | 7.1 ± 0.1 | <0.0001 |
| Paternal affection and support[4] | 10.0 ± 0.1 | 10.9 ± 0.4 | 9.9 ± 0.1 | 0.008 |

[1]Standard error of the mean

[2]A composite score representing parental monitoring (higher score represent lack of parental monitoring)

[3]A composite score representing maternal affection and support (higher score represent lack of maternal affection and support)

[4]A composite score representing paternal affection and support (higher score represent lack of paternal affection and support)

The prevalence of depressive symptoms was higher for females than males, 15.7% and 11.4% respectively. The difference however, was not significant. Over 16% of adolescents who reported depressive symptoms were not living in two-parent households compared to 11.5% of adolescents living with both parents. Self-reported depressive symptoms did not increase with age. The proportion of sexually experienced adolescents reporting depressive symptoms was higher than their sexually inexperienced peers (16.2% vs. 10.8%) and the difference was statistically significant (p=0.04). Adolescents with depressive symptoms had higher mean scores on the parental monitoring scale (6.3 ± 0.2), compared to those without (5.6 ± 0.1). They had higher mean scores on maternal affection and support scale (9.4 ± 0.3) as well as the paternal affection and support scale (10.9 ± 0.4), compared to those without depressive symptoms (7.1 ± 0.1 and 9.9 ± 0.1 respectively). Overall, adolescents with depressive symptoms were more likely to be sexually experienced and to report lack of parental monitoring and supervision, as well as lack of parental (maternal and paternal) affection and support

($p<0.05$). The results of the comparison tests for the variables according to the presence or absence of depressive symptoms are presented in Table 1.

The ORs for all socio-demographic variables remained stable, after adjustment for all socio-demographic and family variables. However, the majority of the variables that were significant at the bivariate level lost their statistical significance when they were included with other covariates in multivariate analysis (Table 2). The result of the final predictive model is presented in Table 3. Adolescents who reported depressive symptoms were more likely to be sexually experienced (OR= 1.59, CI= 1.00-2.52) and more likely to report lack of maternal love and support (OR= 4.06, CI= 2.61-6.32). The proportion of variation in depressive symptoms explained by the overall model is 0.11.

**TABLE 2**
**Unadjusted and adjusted estimates of association of individual and family characteristics of participants with depressive symptoms**

| | Unadjusted (N=748) | | | Adjusted[3] (N=748) | | |
|---|---|---|---|---|---|---|
| Characteristic | OR[1] | 95% CI[2] | p-value | OR | 95% CI | p-value |
| Age in years at time of survey | 1.0 | 0.85-1.21 | 0.60 | 0.9 | 0.77-1.11 | 0.42 |
| Female | 1.5 | 0.92-2.28 | 0.10 | 1.4 | 0.86-2.31 | 0.18 |
| Sexual experience | 1.6 | 1.02-2.51 | 0.04 | 1.6 | 0.99-2.68 | 0.06 |
| Living with both biological parents | 0.7 | 0.44-1.05 | 0.08 | 0.8 | 0.53-1.32 | 0.44 |
| Parental monitoring | 2.0 | 1.33-3.12 | 0.001 | 1.4 | 0.91-2.30 | 0.12 |
| Maternal affection and support | 4.1 | 2.62-6.33 | <0.0001 | 3.5 | 2.16-5.70 | <0.0001 |
| Paternal affection and support | 1.6 | 1.05-1.14 | 0.03 | 1.0 | 0.61-1.50 | 0.84 |

[1]OR: Odds ratio [2]CI: Confidence Interval. [3]Adjusted for all characteristics listed.

**TABLE 3**
**Final model predicting depressive symptoms**

| Characteristic | OR[1] | 95% CI[2] | p-value |
|---|---|---|---|
| Sexually experienced | 1.59 | 1.00-2.52 | 0.05 |
| Lack of maternal affection and support | 4.06 | 2.61-6.32 | <0.0001 |
| Model statistic | | | |
| Nagelkerke $R^2$ | | 0.11 | |

[1]OR: Odd ratio. [2]CI: Confidence Interval.

### DISCUSSION

Based on an extensive literature search, we report that this is the first study to examine the correlates of depressive symptoms among adolescents in a rural setting in Jamaica. Our findings indicated that rural Jamaican adolescents exhibit levels of depressive symptoms similar to those reported in the literature for equivalent groups in the Caribbean and elsewhere with over 14% of adolescents in our study sample affected. This is in agreement with findings of Maharajh et al among Trinidad and Tobago adolescents[20] and adolescents in the United States[2,3,7] – all reporting the prevalence of depressive symptoms among adolescents between 10-35%. More females than males experienced depressive symptoms (15.7% vs.11.4%). This difference did not reach formal statistical significance, contrary to what has been reported in most studies[2,3,7]. However, a couple of other studies have reported no significant gender difference in depression between boys and girls[21,22]. Shek reported no gender difference in his investigation of the reliability and factor structure of the translated BDI among Chinese adolescents[21]. Compas et al also reported no gender differences in depressive symptoms between boys and girls among US adolescents in community samples, finding only statistically significant gender differences in those referred for mental health services[22]. The lack of gender difference in our study may also be due to the relatively smaller number of males compared to females in our study sample.

Lack of perceived maternal affection and support was the most significant finding in our study, associated with depressive symptoms. Adolescents who reported lack of maternal affection and support were more likely to exhibit depressive symptoms, compared to their peers who reported enjoying cordial relationships and support from their mother. Interestingly, the majority of Jamaican households are female-headed[23]. Many parents and children living in single parent households face a lot of social and economic challenges[23]. Living in this type of household likely stresses the adolescent and lack of affection and support by the only parent available may aggravate an already stressful situation, leading to emotional distress. Another explanation could be that depressed adolescents may be less disposed to form supportive relationships with their parents, which could contribute to a perceived lack of parental support. Patten et al reported that depressed adolescents may be less likely to perceive anyone, including their parents, as supportive[2].

An important finding was the tendency of association between being sexually experienced and depressive symptoms. When logistic regression was performed with sexual experience as the only variable the association was very strong. The association became only marginally significant when socio-demographic and family variables were adjusted for. This suggests that socio-demographic and other family context variables may play a role in the association between sexual experience and adolescent depression. Two pathways have been postulated for the association between being sexually experienced and depressive symptoms. It is possible that adolescents' depressive symptoms are occurring in response to sexual activity that took place before they were emotionally ready for it[5]. Regretting early sexual activity is commonly reported by adolescents and supports this hypothesis[24]. This could be true in the Jamaican environment, where early sexual activity among female adolescents has been reported to be coercive[25]. The observed association might also suggest that depressed adolescents may engage in sexual activity as a way of dealing with their emotional problems[7], or as an attempt to seek closeness and acceptance from sexual relationships[5]. This assumption however, was not supported by the longitudinal study conducted by Hallfors et al, who reported that stress associated with sexual activity as playing a causal role in the development of adolescent depressive disorders rather than the participation in sexual activity being a reaction to depression[6].

Several of our measures failed to distinguish between adolescents who reported depressive symptoms and those who did not. Our data suggest that adolescents not living in intact two-parent households tend to have a slightly higher prevalence of depressive symptoms. However, the proportion did not differ significantly from those living in the same household with their biological parents. Similar findings have also been reported in the literature[2]. Relationships found in previous studies[3,7], between increasing age and depression was not obvious in our study. This may be due to the small number of participants below the age of 16 in our study, and the overall moderate sample size. It may also suggest cultural differences in the risk for depression during adolescence. Lack of parental monitoring and lack of paternal affection and support predicted depressive symptoms at the bivariate level but was not predictive in the multivariate analysis. We suggest caution should be exercised in interpreting these non-associations — the findings may be due to insufficient power or small cell samples rather than actual absence of a difference. A larger study with adequate power to detect these differences is suggested.

### Limitations, Implications, and Future Directions

There are several limitations to this study that need to be remarked upon. We cannot establish a causal link between our findings and depressive symptoms because of the cross-sectional design of our study. Further studies are needed to examine the temporal relationship between depressive symptoms and associated risk factors in adolescents. Data for this study were obtained through self-report. While efforts were made to ensure anonymity, the sensitive nature of some of the questions could have resulted in inaccurate responses from the participating adolescents. This study is also limited by the fact that both parental affection and support measures and measures of depressive symptoms were provided by the adolescent. Future research that directly interviews the parents is required to further validate these findings. Non-response to some questions and the missing data generated are also limitations. The statistical replacement of missing values by using means minimized the effect of power lost due to list wise deletion, but reduced variability and introduced bias. This study was restricted to high school students in the rural parish of Hanover, Jamaica. We might have underestimated the prevalence of depressive symptoms, as depressed adolescents are likely to drop out of school. The generalization of the present findings to all adolescents in Jamaica may be limited.

To keep this survey at a manageable level and to ensure appropriate response, we used a short form of the BDI-II to assess depressive symptoms in our study. This could raise possible concerns about the validity and reliability of these measures. However, to avoid bias due to normal mood changes in adolescence we dichotomized depressive symptom score at the upper quartile. It is possible that our measure is not a valid assessment of depressive symptomatology in this population. Nevertheless, the response of adolescents to questions on these salient symptoms of depression indicates the need for further research. We suggest that a larger study should be conducted using the full 21-item BDI-II, or culturally adapted version, to assess depression among Jamaican adolescents.

The strength of this study lies in its being the first epidemiological study of depressive symptomatology among rural Jamaican adolescents. It makes contribution to the literature by simultaneously investigating the contribution of sexual experience and family context variables along with other socio-demographic variables, and allowing for the examination of the relative and independent contributions of these variables to elevated depressive symptoms. Finally, our multivariate model accounted for only 11% of the variations in depressive symptoms in this population, we recognized that we have not controlled for all factors associated with depressive symptoms. The influence of other factors such as age at puberty, illicit drug use, self esteem, religiosity, and other unexamined covariates need to be studied.

In conclusion, our findings support the hypothesis that sexual experience is associated with depressive symptoms only when sexual experience was used as the only explanatory variable for depressive symptoms. There was strong association with lack of maternal affection and support. These findings have implications for prevention of future depression. As most homes are female-headed, establishing support systems for the mother to take care of their adolescent children may decrease the odds of depressive symptoms. Sexually experienced adolescents may require screening for depression. Adolescent health practitioners should consider these factors when designing prevention programs. Depressed adolescents may actually benefit from family and cognitive behavior therapy rather than drugs. Finally, we encourage further research to fully explore all factors that could predispose Jamaican adolescents to depression.

### ACKNOWLEDGEMENTS

This work was supported by the Minority International Research Training Grant (#T37-TW00077) awarded to the last author (Dr. Pauline Jolly) by Fogarty International Center, National Institute of Health, and the Ministry of Health, Jamaica. The authors of this article take responsibility for its contents and not the National Institute of Health or the Ministry of Health, Jamaica. The authors would like to thank the principals and students of the schools in the parish of Hanover, Jamaica that participated in the study.
